# The clinical significance of circulating tumor cells and T lymphocyte subtypes in pancreatic cancer patients

**DOI:** 10.1080/21655979.2021.2023800

**Published:** 2022-01-16

**Authors:** Yasi Xing, Xinfa Zhang, Fangyuan Qin, Jingwen Yang, Lei Ai, Qingsong Wang, Yaping Zhai

**Affiliations:** a Henan Eye Institute, Henan Eye Hospital, People’s Hospital of Zhengzhou University, Henan Provincial People’s Hospital, Zhengzhou, Henan, China; b General Surgery, Shandong Provincial Coal Taishan Sanatorium, Taian, Shandong, China; c Department of Clinical Laboratory, Shandong Provincial Coal Taishan Sanitarium, Taian, Shandong, China

**Keywords:** circulating tumor cells, pancreatic ductal adenocarcinoma, metastasis, recurrence, progression-free survival, cellular immunity

## Abstract

Circulating tumor cells (CTCs) are sensitive and reliable biomarkers for tracing relapsed and metastatic cancer. Here, we explore the clinical significance of CTCs and T lymphocyte subtypes in patients with pancreatic cancer. A total of 106 patients with the pancreatic cancer were enrolled in this study. The enrichment and identification of CTCs were achieved before treatment by a PatrolCTC detection technique. Flow cytometry (FACS) was used to characterize CD4, CD8, natural killer (NK) cells, and Tregulatory (Treg) lymphocyte subtypes. Interleukin-2 (IL-2), Interleukin-4 (IL-4), Interleukin-17A (IL-17A), Interleukin-10 (IL-10), and Interferon γ (IFN-γ) were measured by meso-scale discovery (MSD) assay. Among these patients, 44 (41.5%) patients with pancreatic ductal adenocarcinoma (PDAC) were female and 62 (58.5%) cases were male. Case numbers with II–IV tumor-node-metastasis (TNM) stages were 32 (30.2%), 50 (47.2%), and 24 (22.6%), respectively. The positive rate of CTCs before surgery was 37.5% (12/32), 88.0% (44/50) and 100% (24/24) in stage II, III, and IV patients, respectively. Total CTCs, mixed CTCs, and mesenchymal CTCs (MCTCs) were strongly relevant to shorter progression-free survival (PFS) of the patients. In addition, total CTCs (≥6) and positive MCTCs were also significantly correlated with recurrence and metastasis. The patients with high CTCs also had low levels of CD4, CD4/CD8 ratio, NK cells, IL-2, and IFNγ. In contrast, Treg cells had significant elevation in PDAC patients. These results indicated that CTCs number in PDAC patients was an independent indicator for worse PFS. High CTCs also had strong correlation with weak cellular immunity functions.

## Introduction

Worldwide, pancreatic ductal adenocarcinoma (PDAC) is one of the most lethal cancers in adults. Its incidence is almost the same as its mortality rate with less than 6% five-year survival rate [1–3]. Pancreatic cancer is an extremely aggressive disease and has little benefit from chemotherapy or radiation therapy [4]. Surgery resection is no doubt the most effective therapeutic method for non-metastasized and local cancer. However, only 10–20% of patients were considered as resectable surgery because most people are in metastatic stages at their initial diagnosis [5,6]. In addition, most patients with radical resection eventually have quickly developed recurrence or metastases after surgery [7]. Approximately 20% patients experienced recurrence within the first 6 months [8]. So far, no specificity biomarkers are available for the early detection of PDAC. Currently, PDAC diagnosis is based on tumor biopsy and imaging data like computed tomography (CT) or magnet resonance imaging (MRI) [9]. Due to the presence of many stromal cells in cancer tissues, false-negative results are inevitable. High serum CA19-9 may be a good biomarker for monitoring advanced PDAC, but it is also found in benign pancreatic diseases like pancreatitis, acute cholangitis, cirrhosis, and other cancers [10]. Therefore, it is urgent to identify more sensitive and reliable biomarkers for stratification of patients at its early stage to predict prognosis of patients.

Circulating tumor cells (CTCs) are believed to be from primary tumors and are released into blood circulation for tumor metastasis [11]. Many evidences have revealed that epithelial to mesenchymal transition (EMT) mechanism contributed to this process because EMT promotes phenotypic conversion from epithelial to mesenchymal features of tumor cells [12–15]. This process usually promotes tumor cells migration, invasion, and resistance to apoptosis. When CTCs travel from primitive tumor sites to distant organs, they can perform the mesenchymal – epithelial transition process to induce a new metastasis [16]. CTCs are divided into three types: epithelial CTCs (eCTCs), mesenchymal (MCTC), and mixed CTCs based on their surface markers [17]. More studies have indicated that different types of CTCs may be an excellent biomarker for predicting disease progression of many cancers like breast [12], hepatocellular carcinoma [18], ovarian cancer [19], and esophageal carcinoma [20]. In addition, peripheral blood samples from patients are easily obtained at one or multiple time points during the diagnosis and treatment of disease. These advantages have driven CTCs detection in the PADC patients to get great benefits for the prognosis of patients.

So far, reports of biomarkers for predicting prognosis in patients with pancreatic cancer are limited. A few studies have shown that CTCs in PDAC patients may be a valuable biomarker in predicting the outcome and tumor biology of PDAC [21–23]. A meta-analysis indicated that the pancreatic patients with positive CTCs had a poorer PFS and overall survival (OS) than CTC-negative patients [24]. In addition, recent studies indicated that cellular immunity dysfunctions were critical factors in tumor genesis [6,25–27]. T lymphocyte is divided into CD4 and CD8 subtypes according to its functions and surface markers [28]. CD4 T lymphocyte further characterized into Th1, Th2, Th17, and T reg with specific transcription factor and secreting cytokines [29,30]. Here, we investigated profiles of CTCs and T lymphocyte subsets in the peripheral blood obtained from patients with pancreatic cancer. We hypothesized that CTCs, CD4, CD8, NK cell, and Treg cell number in PDAC patients are strongly associated with the prognosis of disease. To address this hypothesis, we aimed to measure CTCs as well as T lymphocyte subtypes and evaluated PFS in PDAC patients. Our goal was to explore the relationships between CTCs or T lymphocyte subsets and the prognosis in patients with pancreatic cancer. These results will guide the prediction of prognosis in the PDAC patients.

## Materials and Methods

### Patient samples

We retrospectively reviewed 106 PADC patients with well-established tumor, node, and metastasis (TNM) staging who underwent diagnosis from January 2017 to October 2020 in Henan Provincial People’s Hospital. Their diagnoses were confirmed by clinical pathologists using biopsy samples combined CT or MRI images data. For CTCs identification and T lymphocyte subset profiles, 10 milliliter (mL) peripheral blood samples were obtained from all patients as well as control before treatment. The follow-up of patients was started at 3 months after treatments, and then repeated at every 3 months in the first year. The final cutoff time was one year after CTCs detection. This study protocol was reviewed and approved by the review board and ethics committee of Henan Provincial People’s hospital. Written informed consent was obtained from all participants before our study.

### Characterization of CTCs via the CanPatrol System and Tricolor RNA-ISH Method

The isolation and characterization of CTCs in the PDAC patients and control has been followed by previous description [31]. Briefly, after the patients were confirmed for PDAC diagnosis by pathologist, 5 mL peripheral blood was immediately taken and transferred into ethylenediaminetetraacetic acid (EDTA)-coated tubes, which were spun for 30 minutes at 1500 revolutions per minutes (RPM) by Ficoll 400 density gradient liquid (GE healthy,USA).The plasma was collected and stored at −20°C for cytokines assay. The remaining cells were further separated by CanPatrol CTC enrichment technique (SurExam, Guangzhou, China).

**Figure 1.**
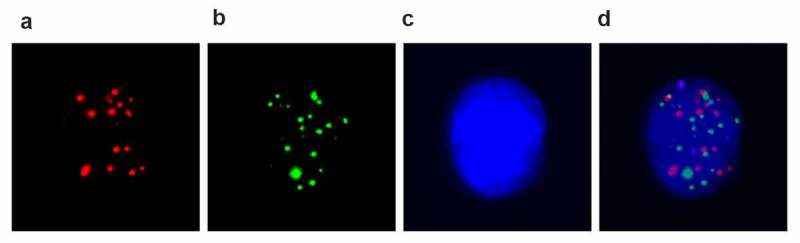
**EMT phenotypes of CTCs were detected by the RNA in situ hybridization in renal cancer patients**. A: Fluorescence microscopy images show three types of CTCs with positive expression of epithelial markers (EpCAM and CK8/18/19, red dots); B, mesenchymal markers (Vimentin and Twist, green dots); C, DAPI stained nuclear; D, biphenotypic markers; Pictures were taken in immunofluorescence microscope by 40x magnification. CTC, circulating tumor cells; DAPI, 4′,6-diamidino-2-phenylindole (DAPI).

For identification of different CTCs subtypes, cells were incubated with Alexa Fluor 594 conjugated epithelial markers EpCAM, CK8/18/19, Alexa Fluor 488 conjugated mesenchymal markers vimentin and Twist, and 4′,6-diamidino-2-phenylindole (DAPI) stained nucleus, respectively. After staining 30 minutes at 4°C, cells were washed with 2% serum PBS solution and taken pictures in immunofluorescence microscope. The representative pictures are shown in Figure 1).

### T lymphocyte subset measurement by flow cytometry

T lymphocyte subset analysis was performed in the described procedure [32]. Briefly, peripheral blood mononuclear cells (PBMCs) were isolated in the above procedure. PBMCs were blocked with FcR and live dye was incubated for 15 minutes. Cells were washed twice with FACS buffer. Firstly, anti-Foxp3 antibody was incubated for 30 minutes at 4°C in intracellular staining reagents, and then washed twice with FACS buffer. For surface markers staining, anti-human CD4, CD8, CD14, CD16, CD56, and CD25 antibodies were added into cells and incubated for 30 minutes at 4°C. Cell analyses were performed Fortassa FACS machine (BD pharmingen, USA). Cell populations were analyzed by flow jo software (version 10.7.1, Tree Star, Ashland, OR,USA).

### Cytokine levels were detected by MSD assay

To assess cytokines levels in PDAC patients, we measured IL-2, IL-4, IL-17A, IL-10, and IFN-γ levels following the published method [33]. Above frozen serum was thawed at room temperature. Each sample was diluted at 1:20 ration by dilute2 solution. Standard curve reagents and procedures were followed by introduction by manufacturer (V-PLEX Plus proinflammatory Panel1 human kit, Cat#:K15049G, MSD, Rockville, Maryland, USA). The results were analyzed using MSD benchwork software.

### Statistical analysis

The association of CTC levels and clinicopathological characteristics were analyzed by the chi-square test. Comparison of cytokine levels was performed using two-tailed Student T-test. PFS was calculated by the Kaplan–Meier method and compared with the log-rank test. Analyses were performed in Graphpad prism 9.0.software. All two-sided *p*-values less than 0.05 were considered to be significant.

## Results

### Patient characteristics

To investigate the relationship between CTCs and clinical characteristics of PDAC patients, we firstly presented their clinical characteristics. A total of 106 PDAC patients with TNM staging and 10 cases of pancreatitis were enrolled in this study. The clinicopathological features of the patients are shown in Table 1, including age, gender, TNM staging, and CTCs count. The results indicated that less 60-year-old cases accounted for 31% (33/106) and more than 60-year-old patients were 69% (73/106). Female was 41.7% (44/106) and male was 58.3% (62/106). II, III, and IV TNM staging cases were 32 (30.6%), 50 (47.2%), and 24 (22.3%), respectively. Interestingly, CTCs positive rate was significantly increased with advanced TNM staging. This result revealed that CTCs positive rate was not relevant to age and gender of PDAC patients, but was strongly associated with TNM staging of patients.

**Table 1.** Relationship between the presence of circulating tumor cells (CTCs) and the clinical features of pancreatic carcinoma

	Number of Cases	CTC-positive(%)	CTC-negative (%)	χ2	P values
Age					
≤ 60 year-old	33 (31%)	23 (69.2%)	10 (30.8%)	1.66	0.20
>60 year-old	73 (69%)	64 (87.0%)	9 (13.0%)		
Gender					
Female	44 (41.7%)	32 (80.0%)	12 (20.0%)	0.005	0.94
Male	62 (58.3%)	50 (81.0%)	12 (19.0%)		
Pathological Stage					
II	32 (30.6%)	12 (36.3%)	20 (63.7%)	19.63	0.0002
III	50 (47.2%)	44 (88%)	6 (12.0%)		
IV	24 (22.3%)	24 (100%)	0 (0.0%)		

### Profiles of CTC subtypes in the PDAC patients

To further validate CTCs features in different stagings of PDAC patients, we characterized the relationship between CTC subtypes and staging of PDAC patients. CTCs were identified in peripheral blood from 106 patients and 10 control cases via Canpatrol technique and tricolor RNA-ISH method. CTCs were divided into based on their surface markers. The epithelial CTCs have positive EpCAM and CK8/18/19 expression. MCTCs have positive vimentin and Twist. In addition, all cellular nucleuses were labeled with DAPI staining. The epithelial CTCs, MCTCs, and mixed CTCs can be distinguished by different immunofluorescence dye staining (Figure 1). In the present study, total 26 patients had not detected CTC including 20 staging II and 6 staging III. All 24 staging IV patients had positive CTCs. No CTCs were detected in all pancreatitis patients. Among different age and gender, no significant differences were found in CTC positive and negative cases (Table 1). In contrast, all 24 staging IV patients had positive CTCs and were significantly higher staging II (P = 0.0002). This result confirmed that CTC positive rate was dramatically relevant to staging of PDAC patients.

### The association between CTCs and patients’ prognosis

**Figure 2.**
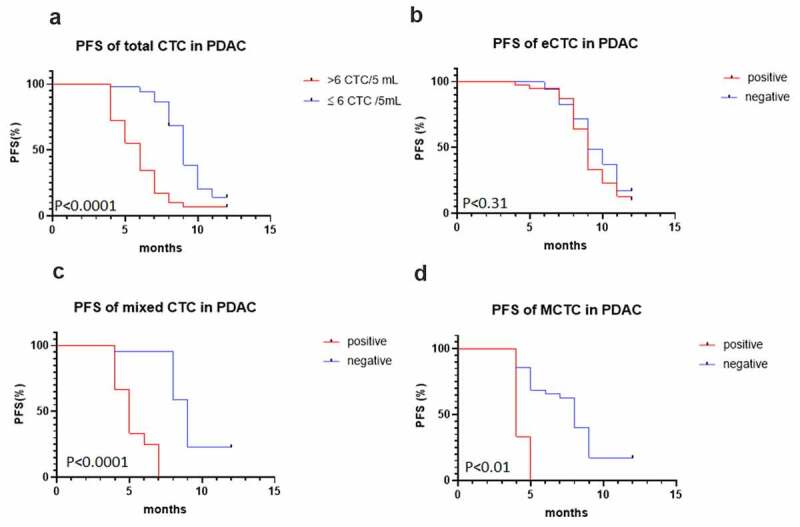
**Kaplan–Meier curves for progression-free survival (PFS) of patients according to CTC, epithelial CTCs, mixed CTCs, and mesenchymal CTC (MCTC)**. (a) total CTC; (b) epithelial CTCs; (c, d) Mixed MCTC and MCTC with PFS. CTC, circulating tumor cells; PFS, progression-free survival; MCTC, mesenchyaml CTC.

To assess the clinical significance of CTCs in predicting the prognosis of the PDAC patients, we compared the PFS of the patients with median number of total CTC counts (CTC >6 or CTC ≤6), mixed CTCs, and MCTCs, respectively. Of the 80 patients with positive CTCs cells, 29 (36.1%) had CTCs > 6 and 51 (63.9%) had CTC ≤ 6 at baseline. The follow-up duration of all patients was 12 months after the initial diagnosis. In total, only 11 patients (13.9%) patients had not experienced a clinical relapse or metastasis by the end of follow-up. The Kaplan–Meier’s survival curves revealed that patients with total CTCs > 6 had significantly poorer PFS (*P* < 0.001) than those with total CTC≤ 6 (Figure 2). Interestingly, more than 6 CTCs/5 mL peripheral blood in total CTCs (Figure 2A, P < 0.0001), mixed CTCs (Figure 2C, P < 0.0001), and MCTC (Figure 2D, P < 0, 01) had poorer PFS than epithelial CTCs (Figure 2b, p = 0.31). In a multivariate Cox regression analysis, we found more than 6 total CTCs (hazards ration [HR], 7.2, 95% confidence interval [CI],3.56–14.70, *P* = 0.0001), mixed CTCs (HR, 5.31, 95% CI, 1.71–16.45, *P* = 0.0001), MCTC (HR 4.14, 95%CI 0.44–39.0, *P* = 0.01) (Table 2). These data indicated that high total CTCs number in total CTCs, mixed CTCs, and MCTC had significantly higher risk of progression compared with those epithelial CTC.

**Table 2.** Comparison of PFS in different CTC cell number

Variables	PFS in >6 CTC (months)	PFS in ≤6 CTC (months)	HR	% 95 CI	P value
Total CTC	5	9	2.96	1.13 to 7.75	0.0001
eCTC	4	8	1.70	0.38 to 7.5	0.12
MixedCTC	5	9	5.31	1.71to16.45	0.0001
MCTC	4	8	4.14	0.44 to 39	0.01

PFS, progression-free survival; CTC, circulating tumor cell; eCTC, epithelial circulating tumor cell; MCTC, mesenchymal circulating tumor cell.HR, hazard ratio; CI, confidence interval.

### Alternations of T lymphocyte subsets in late-stage PDAC patient

To investigate cellular immunity function in PDAC patients, we carried out T lymphocyte subtypes analysis in PDAC patients. The results are shown in Table 3. Compared to pancreatitis patients, CD3, CD4, and NK cells percentage were dramatically declined in PDAC patients (P = 0.0001). In contrast, CD8 had no big differences between control and PDAC patients (P = 0.13). In addition, Treg cells in PDAC patients were significantly increased than control (p = 0.0003). These results revealed that T lymphocyte was dysfunctional in PDAC patients.

**Table 3.** Association between PDAC and different subtypes of T lymphocyte (mean ± SD)

Variables (%)	Control (n = 10)	StageII (n = 32)	Stage III (n = 50)	Stage IV (n = 24)	F value	P value
CD3	76.67 ± 5.16	60.07 ± 10.26	55.06 ± 13.15	53.2 ± 7.82	10.83	0.0001
CD4	42.47 ± 4.89	30.18 ± 7.36	22.68 ± 7.53	20.05 ± 4.88	13.31	0.0001
CD8	25.37 ± 2.92	23.59 ± 5.88	23.36 ± 7.18	18.75 ± 3.51	6.97	0.13
CD4/8	1.66 ± 0.24	1.31 ± 0.31	1.07 ± 0.53	1.07 ± 0.20	5.27	0.003
NK cells	20.05 ± 2.90	15.47 ± 3.5	14.76 ± 3.27	13.31 ± 2.84	7.15	0.0004
Treg cells	5.1 ± 1.37	9.4 ± 2.86	9.72 ± 2.72	9.1 ± 2.12	7.89	0.0003

PDAC, pancreatic ductal adenocarcinoma; NK, natural killer; T reg, T regulatory lymphocyte.

### Cytokine levels in late-stage PDAC patient

To further evaluate T cellular immunity function in PDAC patients, we performed IL-2, IL-4, IL-17A, IL-10, and IFN-γ measurement by MSD assay. The results showed that IL-2 and IFN-γ levels in PDAC patients were significantly declined compared with control patients (P = 0.01 and 0.0001, respectively, Table 4). In contrast, IL-4 and IL-17A had no dramatically changed compared with the control patients (P = 0.37 and 0.42, respectively). Moreover, IL-10 in PDAC patients was markedly higher than in control patients. These data further confirmed that T cell immunity functions were disordered.

**Table 4.** Association between PDAC and cytokines (mean ± SD)

Variables (%)	Control (n = 10)	StageII (n = 32)	Stage III (n = 50)	Stage IV (n = 24)	F value	P value
IL-2	16.35 ± 4.15	10.09 ± 1.47	9.71 ± 5.66	8.19 ± 3.63	6.46	0.001
IL-4	10.69 ± 5.82	7.77 ± 3.01	8.36 ± 4.01	7.68 ± 2.59	1.073	0.37
IL-17A	7.56 ± 2.85	9.27 ± 3.59	7.17 ± 2.77	7.8 ± 3.30	0.96	0.42
IL-10	23.88 ± 4.64	30.41 ± 3.84	30.24 ± 3.06	36.55 ± 6.39	10.56	0.0001
IFN-γ	8.73 ± 1.75	4.69 ± 1.64	4.74 ± 1.68	1.46 ± 1.05	29.30	0.0001

IL-2, interleukin-2; IL-4, interleukin-4; IL-17A, interleukin-17A; IL-10, interleukin-10; IFN-γ, interferon γ.

## Discussion

PDAC disease is a very aggressive cancer because most patients are their advanced staging when they were diagnosed. So far, no specific biomarkers are available for the diagnosis of PDAC patients at its early staging. Recently, many studies have shown that CTC is a critical biomarker for tracing metastatic cascades and predicting the prognosis of cancer patients [13,14–15,34–36]. As for pancreatic cancer, several published studies have also focused on CTCs in advanced staging with various techniques [37–39]. In the present study, we detected total CTCs, mixed CTCs, and MCTCs with pancreatic cancer and found that these CTC subtypes had a significant clinical association with PDAC progress predication. We also found that T lymphocytes in PDAC patients were dysfunctional.

As mentioned previously, CTCs in the bloodstream can be classified into three types, and their detection relies on a combination of membrane filtration and epithelial/mesenchymal biomarker-based identification. Recent reports indicated that the expression of EMT markers in CTCs is a relevant process for invasion and metastasis in several cancers, including breast, colorectal, non-small cell lung, gastric, and prostate cancers [40–43]. The CTCs detection method in the present study allowed the isolation of pivotal EMT CTCs in pancreatic cancer. Total CTCs and MCTCs cluster detection could contribute to improving the accuracy and clinical implications of CTCs. We initially found that positive CTCs were not relevant to age and gender. However, positive CTCs were strongly associated with T staging. Similar result was also found in non-metastatic breast cancer, where preoperative CTCs were poorly associated with tumor size, grade or lymph node status [44]. However, CTCs appear to provide important reference information regarding an individual patient’s risk for relapse or progression. In the present study, patients with higher MCTCs were more likely to have a bad clinical outcome during follow-up. More than six total CTCs, mixed CTCs, and higher positive MCTCs were independent prognostic indicators for poorer PFS.

Recent clinical studies showed that T cell dysfunction in cancer patients was a critical factor for cancer progress [26,27]. T lymphocyte is divide into different subsets such as Th1, Th2, Th17, and Treg according to their specific transcription factor and secreting cytokines. These subtypes play extremely critical roles in regulating human physiology process and response to treatment [45–47]. Indeed, our current data indicated CD3, CD4, and NK cells were significantly declined in PDAC patients. In contrast, Treg cells were dramatically increased. IL-2 and IFN-γ were greatly decreased. These results confirmed that T cell function was important for PDAC progress.

In addition to the above T lymphocyte subtype changes in the peripheral blood of PDAC patients, recently, many studies revealed that tumor-associated lymphocytes (TALs) and tumor-infiltrating lymphocytes (TILs) are closely relevant to progress and response for immunotherapy in cancer patients [48–50]. In the tumor microenvironments (TMEs), there are distinct TALs and TILs subtypes in different cancer types. They determined the disease progress and response to treatments. Therefore, analyses of TALs and TILs of tumor microenvironments in PDAC patients will have great benefits for the therapy of PDAC patients in the future.

## Conclusion

The current study demonstrated that total CTC (CTC > 6), positive mixed CTCs, and positive MCTCs before treatments were significantly shorter PFS. The PDAC patients had low CD4, CD4/CD8 ratio, NK cells, and high Treg cells, indicating that cellular immune functions were impaired.

## Data Availability

Data is available from the corresponding author on request.

## Research highlights

- CTC is a biomarker for monitoring the outcomes of PDAC patients.
- High CTCs, mixed CTCs, and MCTCs in PDAC patients had shorter PFS.
- The patients with high CTCs had low CD4, NK cells, IL-2, IFNγ, and high Treg cells.
